# Study on Ultraviolet Aging Mechanism of Carbon Nanotubes/SBS Composite-Modified Asphalt in Two-Dimensional Infrared Correlation Spectroscopy

**DOI:** 10.3390/ma14195672

**Published:** 2021-09-29

**Authors:** Xuewen Zheng, Wenyuan Xu, Shuangrui Xie

**Affiliations:** 1 School of Civil Engineering, Northeast Forestry University, Harbin 150040, China; 352613703@nefu.edu.cn (X.Z.); shuangruixie2021@126.com (S.X.); 2 Harbin Municipal Engineering Design Institute, Harbin 150040, China

**Keywords:** ultraviolet aging mechanism, carbon nanotubes, SBS, asphalt binder, two-dimensional infrared correlation spectrum

## Abstract

In order to explore the influence mechanism of carbon nanotubes on the ultraviolet (UV) aging properties of the SBS-modified asphalt binder, the changes of functional groups in the one-dimensional infrared spectrum and two-dimensional infrared correlation spectrum are studied in this paper. The results show that the UV aging process of the SBS-modified asphalt binder is the process of alkane chain cleavage and reorganization, the formation of oxygen-containing functional groups and decomposition of SBS. The incorporation of carbon nanotubes can reduce the mutual conversion of methyl and methylene functional groups, inhibit the decomposition of butadiene and the destruction of C = C double bonds in SBS. The degradation of SBS during the process of UV aging leads to the change of many functional groups and acceleration of the aging of the SBS-modified asphalt binder. The addition of carbon nanotubes can effectively alleviate the degradation of SBS and the formation of oxygen-containing functional groups at the early stage of UV aging, and reduce the influence of these two changes on other functional groups; thus, improving the anti-aging performance of the SBS-modified asphalt binder.

## 1. Introduction

Carbon nanotubes (CNTs), as a new type of nanomaterial, have been widely used in various fields since they were discovered by Japanese scientist Iijima S. in 1991 [1,2,3], and many research results have also been achieved in road research [4,5,6]. In recent years, CNTs have been used to enhance various properties of asphalt binder. Melo [7] et al. found that CNTs can enhance the high temperature rheological properties of asphalt binder and the rutting resistance of asphalt mixture. Subsequently, his team shows that adding 2% MWNTS to the asphalt binder significantly improved the asphalt mixture’s resistance to deformation and fatigue [8]. Amin [9] et al. investigated the effect of multi-walled carbon nanotubes on asphalt binder performance in view of the climate in Egypt. The research shows that multi-walled carbon nanotubes can improve the high and low temperature performance and anti-aging performance of asphalt binder. Faramarzi [10] et al. compared the mechanical properties of virgin asphalt binder and virgin asphalt binder doped with CNTs, and the study show that CNTs could significantly enhance the resilient modulus, and creep the behavior and fatigue performance of virgin asphalt binder. Similar research conclusions also appeared in the study of AMERI et al. [11]. In addition, the research of Karahancer et al. [12] also shows that CNTs can improve the water damage resistance of asphalt binder. The results of Mansourkhaki et al.’s research show that 0.5% and 1.2% CNTs have a positive effect on the low temperature performance by improving the thermal cracking resistance, and the asphalt mixtures with 0.5% and 1.2% CNTs show a higher resistance against crack growth than the unmodified asphalt mixture [13].

SBS-modified asphalt binder has been widely used in asphalt pavement because of its excellent road performance. However, it has two obvious drawbacks; one is its poor compatibility with asphalt binder, and the other is degradation after aging. Fortunately, the research of Aff. M. ASCE et al. suggested that CNTs can not only improve the physical and rheological properties of SBS-modified asphalt binder, but also improve the storage stability of the SBS and asphalt binder [14]. Haq et al. Demonstrated wet mixing techniques better help in achieving the homogeneous dispersion of CNTs in bitumen as compared to dry mixing [15]. In Wang’s paper, the high-speed shear mixing method combined with ultrasonic dispersion technology was adopted to solve the problem of the dispersion of nanomaterials in asphalt binder and the asphalt binder samples were prepared by the melt-blending method. In addition, his research also illustrated that CNTs could reduce the attenuation of physical properties and improve the aging resistance of pure asphalt binder [16]. Wang et al. believed that CNTs could reduce the degradation of SBS during the aging process, and the oxygen-uptake amount of blends combined with SBS and CNTs was lower than that of neat SBS modifiers, demonstrating its important effect on the anti-ageing properties of the SBS-modified asphalt binder [17].

The current research on UV aging can be divided into macro and micro perspectives. On the macro level, it is an evaluation of various performance indicators of the aged asphalt binder. From the microscopic point of view, most of them currently use infrared spectroscopy, scanning electron microscope, fluorescence microscope, atomic force microscope and other methods [18,19,20,21,22,23]. Except for infrared spectroscopy, most of the microscopic experiments are to study the microscopic surface morphology of asphalt binder after aging, and rarely involve the transformation of microscopic molecular chains. The one-dimensional infrared spectroscopy can only be used for the qualitative analysis of microscopic functional groups, and it is difficult to establish a relationship between the changes of functional groups and their mutual effects under different conditions.

Based on the one-dimensional infrared spectrum, the two-dimensional infrared correlation spectrum introduces dynamic variables, and it is more inclined to consider the changes in the functional groups inside the substance and the relationship between the changes in the functional groups under the dynamic variables, which can better analyze the microscopic situation of the asphalt binder after UV aging. Thus, two-dimensional infrared spectroscopy provides a new idea and method for studying the aging mechanism of road materials [24,25,26]. On the basis of previous studies, the UV aging mechanism of the CNTs/SBS composite-modified asphalt binder (CSCMAB) was studied by establishing two-dimensional infrared correlation spectroscopy.

## 2. Materials and Methods

### 2.1. Materials

The raw materials used were modified asphalt binder with 4% SBS in 90 # virgin asphalt binder. The properties of the modified asphalt binder are shown in Table 1.

Admixture used GM-302 multi-walled carbon nanotubes, and its technical indicators are shown in Table 2.

### 2.2. Methods

#### 2.2.1. Sample Preparation

Due to the particularity of nanomaterials, the agglomeration phenomenon was easy to obtain in the preparation process; therefore, in order to disperse the modifier in asphalt binder as much as possible, the preparation process had to be improved. According to the research on preparation technology of carbon nanotubes, it is not possible to mix the modified asphalt binder well by stirring alone, and the modified asphalt binder should be shear by using high speed shear instrument after stirring. In the experiment, JRJ-300-I (Shanghai Specimen Model Factory, Shanghai, China)-type high speed shear agitation emulsifier was used to prepare modified asphalt binder.

In the oven, the asphalt binder was heated to 160 °C, and the heating plate was preheated to 170 °C. The weighed CNTs were added in batches and mixed with SBS asphalt binder. Due to the very small mass of nanomaterials, the speed of the agitator could not be greater than 50 r/min to avoid a large number of flying powder caused by the high speed rotation of the instrument to form a cyclone. After CNTs were added, it was fully stirred until the surface was smooth before the next addition. After all the CNTs were added, the high speed shear instrument was used to shear for 90 min. After shearing, the CSCMAB was removed and swollen for 60 min at the temperature of 160 °C.

#### 2.2.2. UV Aging Test

The configured CSCMAB was placed in an oven, and the asphalt binder samples were irradiated with 500 W ultraviolet lamp. The ambient temperature was controlled at 20 °C with the oven to simulate the photo-oxygen aging of asphalt pavement. The total irradiation time was 120 h, and samples were taken at a set time interval.

#### 2.2.3. Dynamic Shear Rheology Test

Dynamic shear rheology test (DSR) was used to evaluate the permanent deformation resistance of composite-modified asphalt under high temperature. The initial temperature was determined to be 58 °C, and we increased the temperature to 76 °C in 6 °C increments. The rutting factor (G*/sinδ) and phase angle at each temperature were analyzed. The diameter of sample was 25 mm and the pitch was 1 mm. With strain control mode, the strain was 12%, strain scanning was performed on SBS-modified asphalt binder and CSCMAB to determine the linear viscoelastic interval of asphalt binder (LVE). Each experiment was repeated twice to eliminate randomness and ensure the reliability of the results. CSCMAB samples were prepared by adding 0, 0.2%, 0.5%, 1% and 2% CNTs to SBS-modified asphalt binder.

#### 2.2.4. Infrared Spectrum Test

Fourier transform infrared spectrometer (Spectrum 400) was used for testing. The test wave number range was 4000~400 cm^−1^, the spectral resolution was 4 cm^−1^, and the number of scans was 32. The asphalt binder was dissolved in the carbon tetrachloride solution at a ratio of 5% to prepare a liquid sample, and the sample was dropped on a wafer for testing. OMNIC software was used to process the data and draw the infrared spectrum.

## 3. Results and Discussion

### 3.1. Rheological Properties of CSCMAB

**Figure 1:**
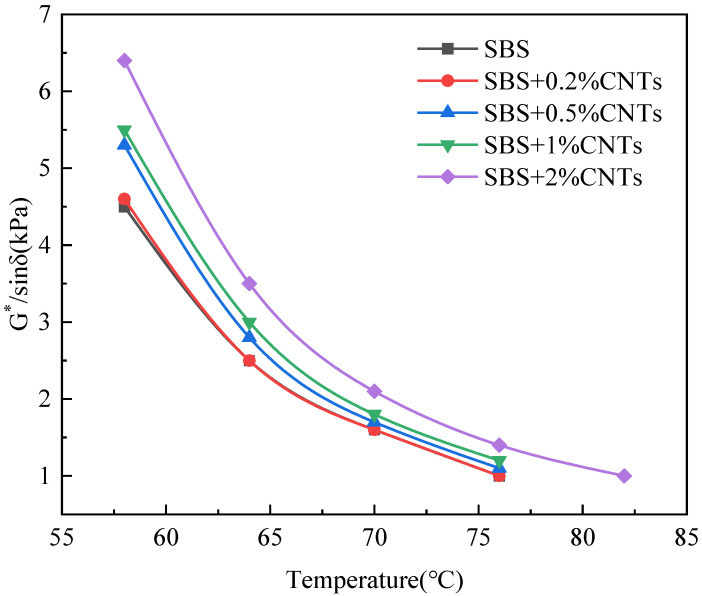
Relationship between G*/sinδ and temperature of unaged asphalt binder.

**Figure 2:**
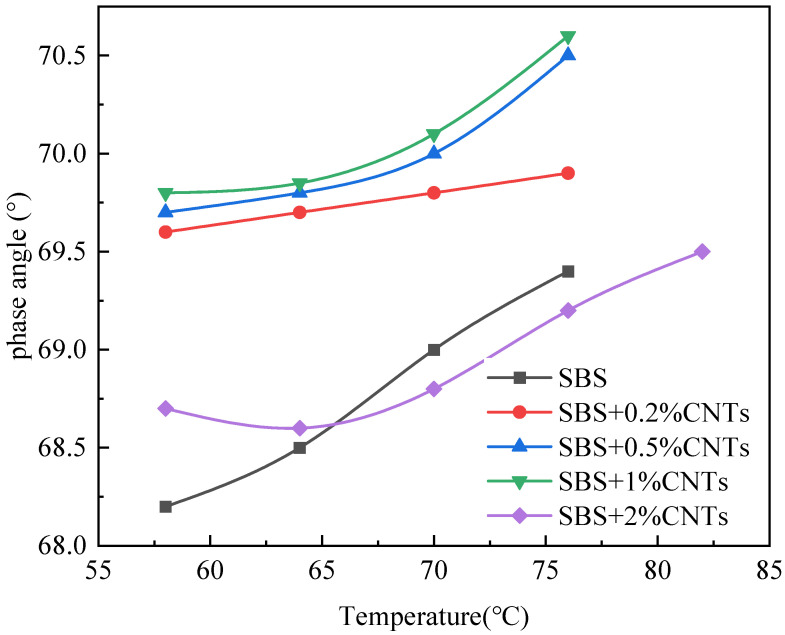
Phase angle of unaged asphalt binder.

**Figure 3:**
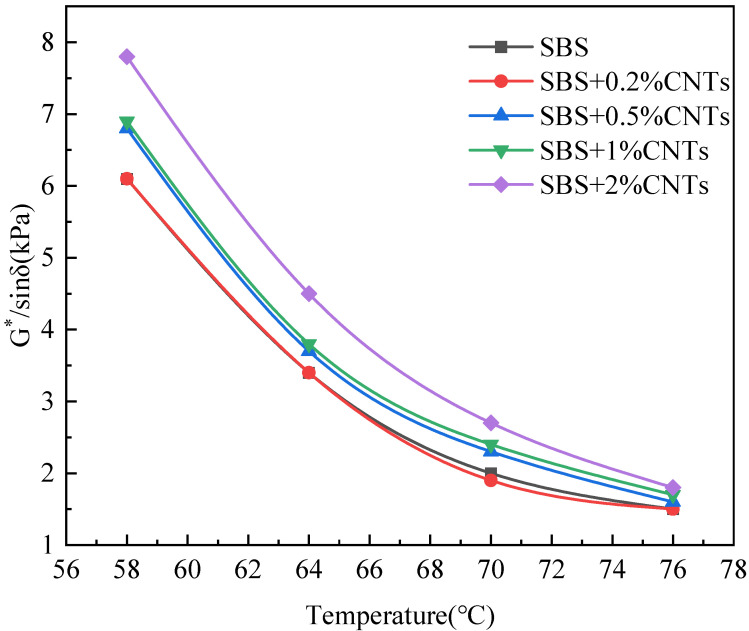
Relationship between G*/sinδ and temperature after UV aging.

**Figure 4:**
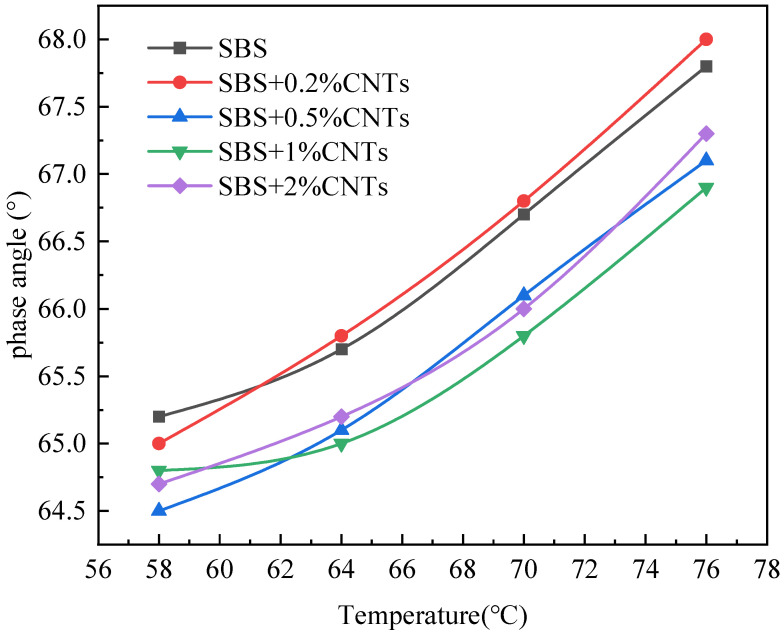
Phase angle after UV aging.

The Superpave specification proposes using the rutting factor (G*/sinδ) and phase angle to evaluate high temperature rheological properties of the asphalt binder. The larger the G*/sinδ is, the better the rut resistance of the asphalt binder [27,28,29]. The phase angle is related to the rheological properties of asphalt binder, and the viscoelastic properties of asphalt binder change with the change of the phase angle. When the phase angle moves to a high level, the asphalt binder changes to a viscous state, and when the phase angle moves to a low level, the asphalt binder changes to an elastic state. In this paper, the SBS-modified asphalt binder without CNTs was used as the control group and CSCMAB as the experimental group to compare the influence of CNTs on the rheological properties of the SBS-modified asphalt binder. Dynamic shear rheological tests were performed on the unaged asphalt binder and the 30 h UV-aged asphalt binder. The results are shown in Figure 1, Figure 2, Figure 3 and Figure 4 [30].

As can be seen from Figure 1 and Figure 3, after UV aging, the rutting factor G*/sinδ of all asphalt binders obviously increased. Comparing the growth rate of the rutting factor, the SBS-modified asphalt increased by 1.314, and CSCMAB with CNTs content from 0.2% to 2% increased by 1.309, 1.286, 1.264 and 1.253, respectively. The growth rate of CSCMAB was lower than the SBS-modified asphalt binder. The results indicated that CNTs could inhibit UV aging of the SBS-modified asphalt binder, and CSCMAB with 2% CNTs had the best effect. It can be seen from Figure 2 and Figure 4 that the phase angles of all asphalt binders gradually increased with the temperature rising, indicating that the asphalt binder changed from an elastic state to a viscous state in the process of heating up. Before UV aging, when the CNTs were added to the SBS-modified asphalt binder, the phase angle of CSCMAB increased gradually at all temperatures with the increase in the CNTs content, indicating that CNTs increased the viscosity of the SBS-modified asphalt binder. However, when the CNTs content increased to 2%, the phase angle of CSCMAB decreased significantly, which was basically equivalent to that of the SBS-modified asphalt binder, indicating that 2% CNTs could inhibit the transformation of the asphalt binder to viscosity and increase the elasticity of the SBS-modified asphalt binder and resist high temperature deformation, and the rate of the phase angle increase in the SBS-modified asphalt binder was significantly higher than that of 2% CSCMAB, indicating that CNTs can help the SBS-modified asphalt binder maintain a certain elastic resistance to deformation and improve the high-temperature performance of the SBS-modified asphalt binder. After UV aging, the phase angle of all asphalt binders decreased. Among several CSCMABS, when the content of CNTs was 2%, the corresponding phase angle curve decreased the least, indicating that 2% CNTs had the best effect on inhibiting asphalt aging and hardening, but the decrease in the phase angle of CSCMAB with 2% CNTs was greater than that of the SBS-modified asphalt binder; however, this did not indicate that the SBS-modified asphalt binder had a better UV aging resistance than CSCMAB, and CNTs also had the contribution of maintaining the asphalt binder’s elasticity. In order to further study the mechanism of CNTs to improve the anti-ultraviolet aging performance of the SBS-modified asphalt binder, SBS + 1% CNTs composite-modified asphalt binder and SBS-modified asphalt binder were prepared as experimental groups. The prepared asphalt binder samples were subjected to UV aging for a total time of 120 h and the aged samples were subjected to an infrared spectrum test.

### 3.2. Infrared Spectrum Test

**Figure 5:**
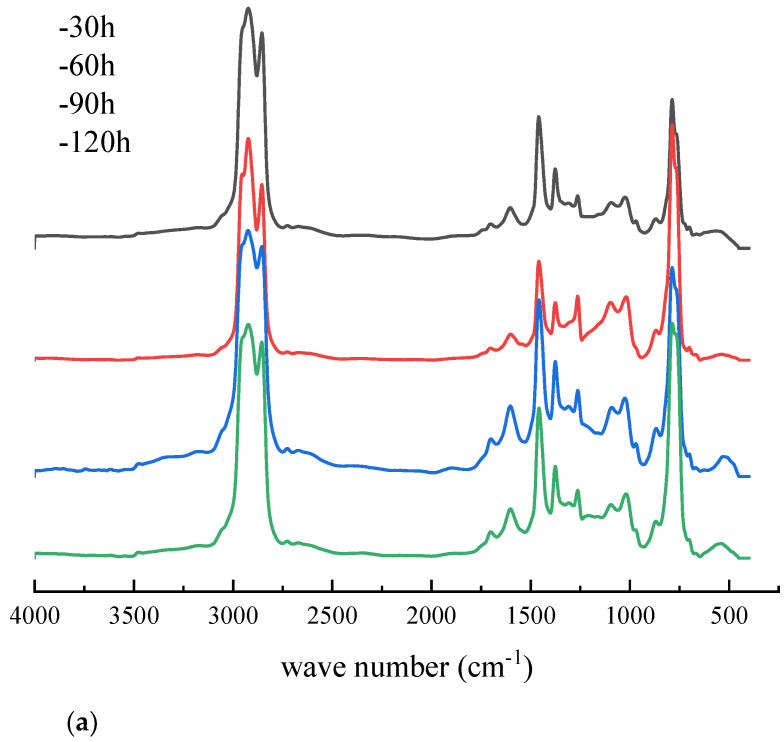

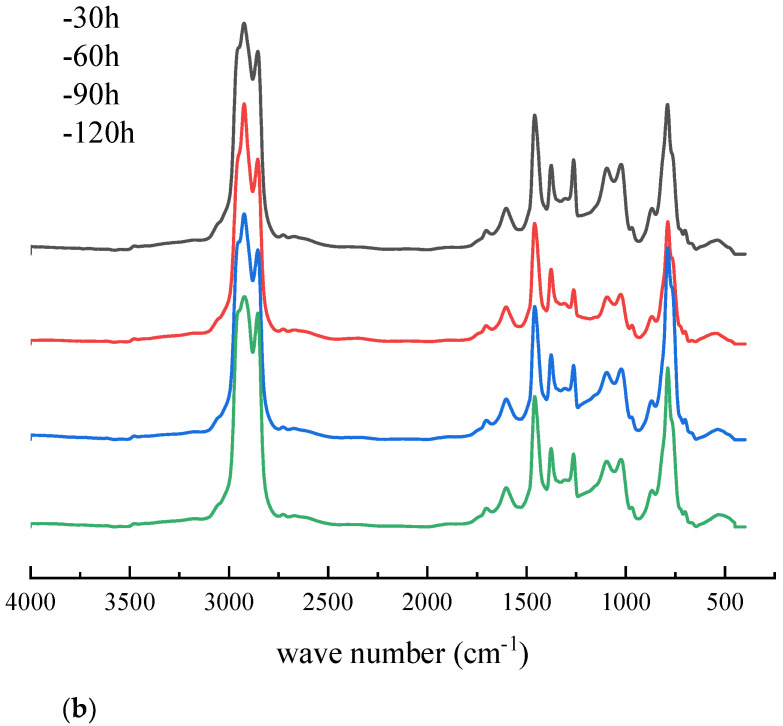
Infrared spectrum of asphalt binders after UV aging. (**a**) SBS-modified asphalt binder; (**b**) SBS + 1% CNTs composite-modified asphalt binder.

According to Figure 5, it can be seen that the asphalt binder had strong absorption peaks at 2923 cm^−1^ and 2853 cm^−1^, which were caused by the asymmetric and symmetrical stretching vibration of methylene-CH2-. The infrared absorption peak at 1603 cm^−1^ was generated by the vibrational absorption of the conjugated double bond C = C on the benzene ring skeleton. The infrared absorption peaks at 1456 cm^−1^ and 1376 cm^−1^ were generated by the bending vibration of methylene and methyl groups. From these two absorption peaks, it could be determined that saturated hydrocarbons were present in the asphalt binder. In the fingerprint region, the 900~650 cm^−1^ region was the out-of-plane bending vibration frequency of the C–H functional group on the benzene ring, which reflected the type of aromatic ring substitution. From this, the presence of aromatic compounds in the asphalt binder could be determined. The infrared absorption peak at 1700 cm^−1^ was caused by the carbonyl (C = O) vibration, and the infrared absorption peak at 1030 cm^−1^ was generated by the sulfoxide (S = O) vibration. These two absorption peaks were characteristic functional groups of asphalt binder aging, reflecting the oxidation of asphalt binder during aging [31,32]. By analyzing the absorption peaks in the infrared spectrum, we could conclude that the asphalt binder was mainly composed of saturated hydrocarbons, aromatic compounds and heteroatom derivatives. By comparing Figure 5a,b, it can be seen that the UV aging process did not add new functional groups to the SBS-modified asphalt binder, and CNTs and the SBS-modified asphalt binder were only physically mixed and a chemical reactions to generate new functional groups did not occur.

### 3.3. Two-Dimensional Infrared Spectrum

#### 3.3.1. Two-Dimensional Synchronous Correlation Infrared Spectrum

Although one-dimensional infrared spectroscopy could determine the types of functional groups in the SBS-modified asphalt binder and CSCMAB, it did not show the changes of functional groups in them. Therefore, based on the one-dimensional infrared spectrum, the two-dimensional infrared spectrum was introduced to further study the effect of ultraviolet aging on the SBS-modified asphalt binder and CSCMAB. The two-dimensional infrared spectrum was obtained by applying a specific perturbation to the sample to induce a dynamic change in the spectral signal, and performing a correlation analysis and calculation on a series of dynamic spectra.

The two-dimensional infrared correlation spectrum can reflect the changes of various components or microstructure units in the sample relative to the external perturbation, and the correlation between these changes [33,34]. In this experiment, the aging time was used as a perturbation variable to study the change of functional groups with the increase in aging time. According to the one-dimensional infrared spectrum, it could be seen that the functional groups of the two types of asphalt binders were mainly concentrated between 1700~450 cm^−1^ and 3000~2800 cm^−1^; therefore, we focused on these two areas.

The two-dimensional infrared correlation spectrum was obtained by using the two-dimensional correlation spectroscopy software independently developed by the State Key Laboratory of Polymer Materials Engineering of Sichuan University.

**Figure 6:**
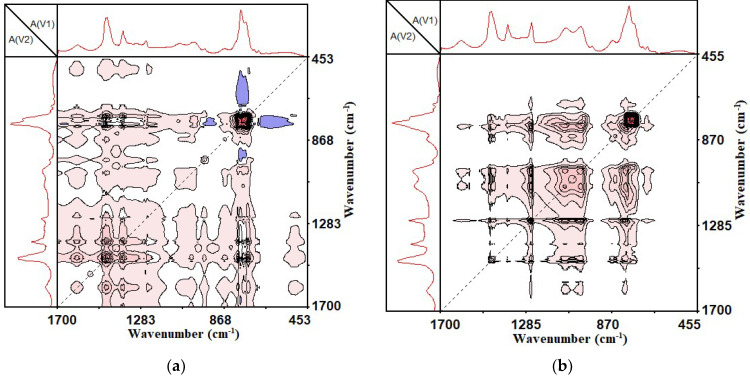
Two-dimensional synchronous infrared correlation spectrum of 1700~450 cm^−1^. (**a**) SBS-modified asphalt binder; (**b**) SBS + 1% CNTs composite-modified asphalt binder.

The automatic peaks located on the diagonal dotted lines indicate the degree of functional group changes as the external perturbation conditions changed. The appearance of the automatic peaks indicates that the functional groups changed as the external conditions changed. The more obvious the automatic peaks, the stronger the changes. As can be seen from Figure 6a, with the increase in the UV aging time, the SBS-modified asphalt had seven automatic peaks in 1700~450 cm^−1^, which were located at 770 cm^−1^, 966 cm^−1^, 1030 cm^−1^, 1261 cm^−1^, 1376 cm^−1^, 1456 cm^−1^ and 1600 cm^−1^. Among them, 770 cm^−1^ was the characteristic peak of styrene in SBS, the automatic peak was the strongest and 966 cm^−1^ was the characteristic peak of butadiene in SBS. This showed that with the increase in UV aging time, the structure of SBS changed greatly, and a significant degradation reaction occurred. The peaks at 1376 cm^−1^ and 1456 cm^−1^ were the bending vibrations of the methyl group and methylene group, indicating that during the ultraviolet aging of the SBS-modified asphalt binder, chain-breaking recombination reactions of alkanes and naphthenes occurred. The peaks at 1030 cm^−1^ and 1261 cm^−1^ were oxygen-containing functional groups in the epoxy compound, indicating that the oxidation reaction occurred during the UV aging. The 1600 cm^−1^ corresponds to the C = C structure on the benzene ring, which was gradually destroyed with the increase in the UV aging time.

It can be seen from Figure 6b that with the increase in UV aging time, CSCMAB had four obvious automatic peaks in 1700~450 cm^−1^, which were located at 770 cm^−1^, 1030 cm^−1^, 1261 cm^−1^ and 1456 cm^−1^. Compared with the SBS-modified asphalt binder, there were no automatic peaks at 966 cm^−1^, 1376 cm^−1^ and 1600 cm^−1^. It showed that the incorporation of CNTs reduced the degradation of butadiene, methylene chain breakage and benzene ring destruction in SBS.

According to previous studies [35,36], the carbonyl group at 1700 cm^−1^ and the sulfoxide group at 1030 cm^−1^ were typical functional groups for the aging of asphalt binder. In the one-dimensional infrared spectrum, absorption peaks of both functional groups also existed; however, in the two-dimensional infrared correlation spectrum, only the automatic peak of the sulfoxide group was seen, and no automatic peak of the carbonyl group was observed. This showed that during the ultraviolet aging of the asphalt binder, the formation of carbonyl groups was not the main cause of aging. The reason why the carbonyl group appeared in the one-dimensional infrared spectrum may have been caused by the thermal oxygen aging during the stirring and heating of the asphalt binder.

**Figure 7:**
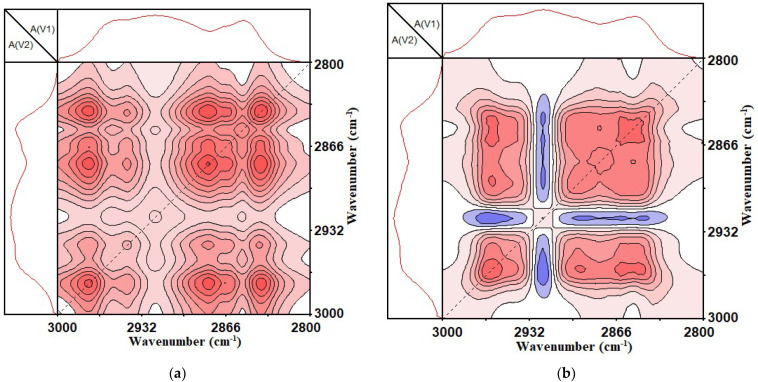
Two-dimensional synchronous infrared correlation spectrum of 3000~2800 cm^−1^. (**a**) SBS-modified asphalt binder; (**b**) SBS + 1% CNTs composite-modified asphalt binder.

From Figure 7, it can be seen that, with the increase in the UV aging time, the SBS-modified asphalt binder and CSCMAB had four obvious automatic peaks in 3000~2800 cm^−1^, which were located at 2838~2853 cm^−1^, 2880~2894 cm^−1^, 2923 cm^−1^ and 2964~2975 cm^−1^. Among them, 2838~2853 cm^−1^ and 2923 cm^−1^ were the symmetrical and asymmetric contraction vibrations of methylene (-CH2-); 2880~2894 cm^−1^ and 2964~2975 cm^−1^ were the symmetrical and asymmetric contraction vibrations of methyl (-CH3).

Compared with the one-dimensional infrared spectrum, it was found that 2880–2894 cm^−1^ and 2964–2975 cm^−1^ did not show a significant peak in the one-dimensional infrared spectrum. This showed that the stretching vibration of the methyl and methylene functional groups in the asphalt binder was difficult to observe in the one-dimensional infrared spectrum due to the weak absorption strength. This also showed that expanding the absorption peak on the two-dimensional scale could improve the resolution of the spectrum, and could distinguish between small peaks and weak peaks that were covered on the one-dimensional spectrum, thereby improving the spectral resolution ability and making up for the limitations of the one-dimensional infrared spectrum.

From the analysis of Figure 7, it can be seen that with the increase in ultraviolet light irradiation time, the content of methyl and methylene in the asphalt binder changed. Therefore, it was inferred that the chain length of the alkane and naphthene in the asphalt binder composition changed with the increase in ultraviolet light irradiation time.

**Figure 8:**
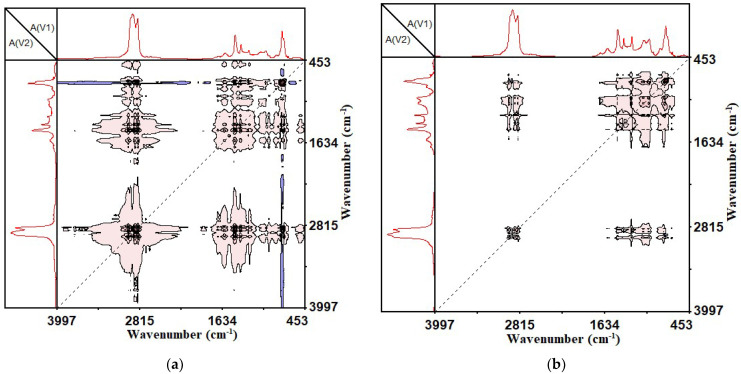
Two-dimensional synchronous infrared correlation spectrum of 4000~450 cm^−1^. (**a**) SBS-modified asphalt binder; (**b**) SBS + 1% CNTs composite-modified asphalt binder.

By comparing Figure 8a,b, it can be seen that, when the degree of change was the same at 770 cm^−1^, the change of other functional groups of the SBS-modified asphalt binder was much larger than those of the CSCMAB. This showed that the incorporation of CNTs could effectively inhibit the functional group changes of the SBS-modified asphalt during the UV aging process, reducing the destruction of the original properties of the UV-aged asphalt binder.

#### 3.3.2. Two-Dimensional Asynchronous Infrared Correlation Spectrum

**Figure 9:**
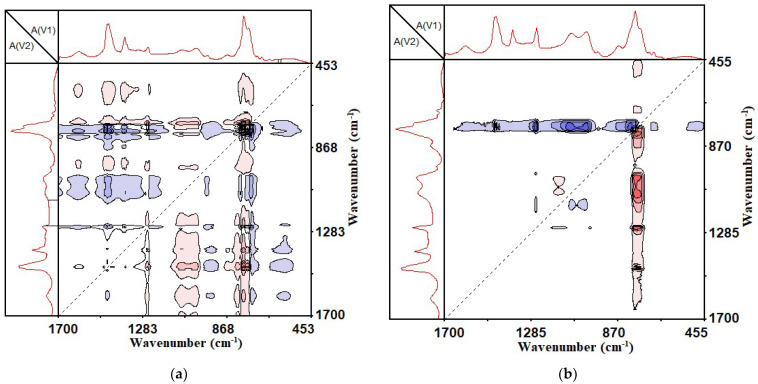
Two-dimensional asynchronous infrared correlation spectrum of 1700~450 cm^−1^. (**a**) SBS-modified asphalt binder; (**b**) SBS + 1% CNTs composite-modified asphalt binder.

**Figure 10:**
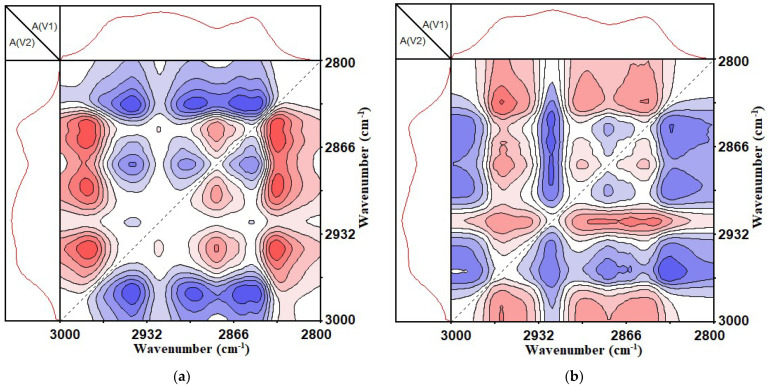
Two-dimensional asynchronous infrared correlation spectrum of 3000~2800 cm^−1^. (**a**) SBS-modified asphalt binder; (**b**) SBS + 1% CNTs composite-modified asphalt binder.

The two-dimensional asynchronous infrared correlation infrared spectrum was different from the synchronous spectrum, and it represented the order of change of the spectral intensity of two dynamic signals. There were no automatic peaks in the two-dimensional asynchronous infrared correlation spectrum, and they were composed entirely of crossed peaks on both sides of the diagonal. The generation of the asynchronous crossover peaks was due to the difference in the intensity of the two spectral peaks. The sign of asynchronous cross peaks could be positive or negative (expressed in shaded colors: red is positive, blue is negative). It can help to identify the sequence of the movement of functional groups of molecules. At the coordinate corresponding to a cross peak (ν1, ν2), if the two-dimensional asynchronous correlation spectrum and the corresponding two-dimensional synchronous correlation spectrum have the same color, it means that the intensity change at ν1 always preceded the intensity change at ν2. When the colors are different, the above rules are just the opposite [37]. The previous analysis of the two-dimensional synchronous infrared correlation spectrum determined which functional groups changed during the UV aging of the CSCMAB. Now, the order of these functional group changes was determined by analyzing the asynchronous spectrum. Among them, Figure 9 is a two-dimensional infrared asynchronous correlation spectrum chart of 1700 to 450 cm^−1^, and Figure 10 is a two-dimensional infrared asynchronous correlation spectrum chart of 3000 to 2800 cm^−1^.

It can be seen from Figure 8a that, as the UV aging time of the SBS-modified asphalt binder increased, almost all functional group changes in the range of 1700~450 cm^−1^ were related to the functional group changes at 770 cm^−1^. According to the rules for determining the sequence of the two-dimensional asynchronous infrared correlation spectrum, it was found that in the two-dimensional asynchronous infrared correlation spectrum of the SBS-modified asphalt binder, the functional groups at 966 cm^−1^ and 1030 cm^−1^ changed earlier than 770 cm^−1^. The functional group at 1376 cm^−1^, 1456 cm^−1^ and 1600 cm^−1^ changed later than 770 cm^−1^. Additionally, in the two-dimensional infrared asynchronous correlation spectrum of CSCMAB, the change of all functional groups were earlier than 770 cm^−1^. According to previous research, the 770 cm^−1^ functional group is the characteristic functional group of styrene in SBS, 966 cm^−1^ is the characteristic functional group of butadiene in SBS, 1030 cm^−1^ is the sulfoxide functional group, 1376 cm^−1^ and 1456 cm^−1^ are a bending vibration functional group of the methyl group and methylene group, and 1600 cm^−1^ is a characteristic functional group on the benzene ring. It shows that, during the UV aging of the SBS-modified asphalt binder, most of the other functional groups would change after the SBS degradation, which explains why SBS could improve the anti-aging performance of asphalt. If the degradation of SBS during UV aging can be reduced in future research, the impact of UV aging on SBS-modified asphalt binder could be greatly reduced. After the incorporation of CNTs, the functional groups at 966 cm^−1^ and 1030 cm^−1^ changed later than 770 cm^−1^. This showed that the addition of CNTs could effectively inhibit the formation of sulfoxide groups and the destruction of butadiene in SBS at the early stage of UV aging, and improve the anti-UV aging ability of the SBS-modified asphalt binder.

According to the foregoing, it can be known that there were mainly changing functional groups between 3000 and 2800 cm^−1^, which were the symmetric and asymmetric functional groups of methyl and methylene, respectively. By observing Figure 10, it is found that within the range of 3000 to 2800 cm^−1^, the functional group changes of the methyl group and methylene group appeared alternately. This showed that during the ultraviolet aging of the two types of asphalt binders, changes such as the fragmentation and recombination of alkanes and naphthenes, the lengthening of short-chain alkanes, and the breaking of naphthenes into chains were observed.

## 4. Conclusions

The following conclusions were obtained by analyzing DSR test results, the one-dimensional infrared spectrum and two-dimensional infrared correlation spectrum of the SBS-modified asphalt binder and CSCMAB after UV aging.

The addition of CNTs can improve the rutting factor, and the greater the addition of CNTs, the greater the increase in the rutting factor, indicating that CNTs can improve the high temperature performance of the SBS-modified asphalt binder. CNTs can delay the increase rate of the phase angle of the SBS-modified asphalt binder with a temperature increase; thus, inhibiting the transformation of the SBS-modified asphalt binder to a viscous state and increasing the elasticity of the SBS-modified asphalt binder to resist deformation. After UV aging, the 2% CNTs had the best effect on inhibiting the SBS-modified asphalt binder aging and hardening.

During the UV aging process, the changes in the SBS-modified asphalt binder and CSCMAB functional groups were mainly the mutual conversion of methyl groups and methylene groups, the generation of oxygen-containing functional groups and the degradation of butadiene and styrene in the SBS structure. The carbonyl group was not the characteristic functional group for the ultraviolet aging of the two asphalt binders. During the UV aging process, the content of carbonyl groups hardly changed, and the largest change in the SBS-modified asphalt was the degradation of SBS and most of the other functional groups would change after SBS degradation. Reducing the degradation rate of SBS in asphalt binder can effectively reduce the effect of UV aging on the asphalt binder. The incorporation of CNTs can effectively inhibit the degradation of butadiene in SBS and the destruction of C = C double bonds in the benzene ring during UV aging. When the degradation level of SBS is the same, the change strength of other functional groups in the asphalt binder is reduced, thereby maintaining the original performance of the asphalt binder. In addition, the incorporation of CNTs can effectively inhibit the formation of oxygen-containing functional groups in the early stage of UV aging, and enhance the UV aging resistance of the SBS-modified asphalt binder. CNTs can effectively inhibit the degradation rate of SBS subjected to ultraviolet aging, so as to maintain the network crosslinking structure of SBS in asphalt binder; thus, enhancing the resistance to the deformation of the SBS-modified asphalt binder and maintaining the elasticity of the SBS-modified asphalt binder. In DSR experiment results, the reaction was shown as an increased rutting factor and an enhanced high-temperature performance. The phase angle of CSCMAB after UV aging was lower than that of the SBS-modified asphalt binder.

## Figures and Tables

**Table 1:** Basic properties of modified asphalt.

Properties	Test Results	Methods
Penetration (25 °C, 0.1 mm)	69.1	JTG E20-2011 T0604
Softening Point (°C)	60.5	JTG E20-2011 T0606
Ductility (5 °C, cm)	33	JTG E20-2011 T0605

**Table 2:** Technical index of GM-302 multi-walled carbon nanotubes.

No.	Test Items		Unit	Eligibility Criteria
1	Pipe diameter		Nm	10–20
2	Tube length		Μm	5–15
3	Purity		%	≥97.5
4	Ash		%	≤2.5
5	Specific surface area		m^2^/g	240–280
6	Tap density		g/cm^3^	0.06–0.09
7	Resistivity		mΩ·cm	80–100
8	Metal content	Fe	Ppm	≤4500
		Co	Ppm	≤4500
		Ni	Ppm	≤100
		Cu	Ppm	≤10
		Zn	Ppm	≤10
		Cr	Ppm	≤10

## Data Availability

The data used to support the findings of this study are included within the article.

## References

[1] Burakova E.A., Dyachkova T., Rukhov A.V., Tugolukov E.N., Galunin E.V., Tkachev A.G., Basheer A.A., Ali I. (2018). Novel and economic method of carbon nanotubes synthesis on a nickel magnesium oxide catalyst using microwave radiation. J. Mol. Liq..

[2] Wang H., Li P., Yu D., Zhang Y., Wang Z., Liu C., Qiu H., Liu Z., Ren J., Qu X. (2018). Unraveling the Enzymatic Activity of Oxygenated Carbon Nanotubes and Their Application in the Treatment of Bacterial Infections. Nano Lett..

[3] Xie J., Ma J., Wu L., Xu M., Ni W., Yan Y.-M. (2020). Carbon nanotubes in-situ cross-linking the activated carbon electrode for high-performance capacitive deionization. Sep. Purif. Technol..

[4] Shuwen Z.H.A.N.G., Jie Z.H.A.N.G., Guichun W.A.N.G., Danying G.A.O. (2019). Effect of Carbon Nanotubes on Thermal Expansion Properties of Cement-based Materials. Chin. J. Mater. Res..

[5] Shi T., Zexin L., Shanshan L. (2019). Autogenous shrinkage and crack resistance of carbon nanotubes reinforced cement based composites. Acta Mater. Compos. Sin..

[6] Zhou X., Zhang X., Xu S., Wu S., Liu Q., Fan Z. (2017). Evaluation of thermo-mechanical properties of graphene/carbon-nanotubes modified asphalt with molecular simulation. Mol. Simul..

[7] Trichês J.V.S.d.M.G. (2016). Evaluation of Rheological Behavior and Performance to Permanent Deformation of Nanomodified Asphalt Mixtures with Carbon Nanotubes (CNTs). Can. J. Civ. Eng..

[8] de Melo J.V.S., Trichês G., de Rosso L.T. (2018). Experimental evaluation of the influence of reinforcement with Multi-Walled Carbon Nanotubes (MWCNTs) on the properties and fatigue life of hot mix asphalt. Constr. Build. Mater..

[9] Amin I., El-Badawy S.M., Breakah T., Ibrahim M.H.Z. (2016). Laboratory evaluation of asphalt binder modified with carbon nanotubes for Egyptian climate. Constr. Build. Mater..

[10] Arabani M., Faramarzi M. (2015). Characterization of CNTs-modified HMA’s mechanical properties. Constr. Build. Mater..

[11] Ameri M., Nowbakht S., Molayem M., Mohammadaliha M. (2016). Investigation of fatigue and fracture properties of asphalt mixtures modified with carbon nanotubes. Fatigue Fract. Eng. Mater. Struct..

[12] Saltan M., Terzi S., Karahancer S. (2018). Performance analysis of nano modified bitumen and hot mix asphalt. Constr. Build. Mater..

[13] Mansourkhaki A., Aghasi A. (2021). Low-temperature fracture resistance of asphalt mixtures modified with carbon nanotubes. Proc. Inst. Civ. Eng.-Transp..

[14] Goli A.A., Ziari H., Amini A. (2017). Influence of Carbon Nanotubes on Performance Properties and Storage Stability of SBS Modified Asphalt Binders. J. Mater. Civ. Eng..

[15] Haq M.F.U., Ahmad N., Nasir M.A., Jamal M.H., Hafeez M., Rafi J., Zaidi S.B.A., Haroon W. (2018). Carbon Nanotubes (CNTs) in Asphalt Binder: Homogeneous Dispersion and Performance Enhancement. Appl. Sci..

[16] Wang R., Yue J., Li R., Sun Y. (2019). Evaluation of Aging Resistance of Asphalt Binder Modified with Graphene Oxide and Carbon Nanotubes. J. Mater. Civ. Eng..

[17] Wang P., Dong Z., Tan Y., Liu Z.-Y. (2016). Anti-ageing properties of styrene–butadiene–styrene copolymer-modified asphalt combined with multi-walled carbon nanotubes. Road Mater. Pavement Des..

[18] Tang N., Yang Y., Fang T., Wang W., Cao S., Pan W. (2018). Aging Behavior of Bitumen Based on Three-Dimensional Fluorescence Spectroscopy. Spectrosc. Spectr. Anal..

[19] Zhang Q., Hou D., Shi J. (2019). Research Progress of Microscopic Characterization of Rubber Asphalt. Mater. Rep..

[20] Jiang W., Li P., Ye W., Shan J., Li Y., Xiao J. (2020). The effect and mechanism of La2O3 on the anti-ultraviolet aging characteristics of virgin bitumen. Constr. Build. Mater..

[21] Liu L., Liu Z., Hong L., Huang Y. (2020). Effect of ultraviolet absorber (UV-531) on the properties of SBS-modified asphalt with different block ratios. Constr. Build. Mater..

[22] Xu J., Li R., Liu T., Pei J., Li Y., Luo Q. (2020). Study on the Effect of Microwave Processing on Asphalt-Rubber. Materials.

[23] Fang J., Tu J. (2019). Effect of ultraviolet (UV) aging on rheology properties and microstructure of polyurethane (PU) modified asphalt. Mater. Res. Express.

[24] Ma D., Liu G., Ou Q., Yu H., Li H., Shi Y. (2018). Discrimination of Common Wild Mushrooms by FTIR and Two-Dimensional Correlation Infrared Spectroscopy. Spectrosc. Spectr. Anal..

[25] Wu F., Zhang Y., Liu W., Zhu N., Chen J., Sun Z. (2020). Comparison of torrefied and lyophilized Dendrobii Officinalis Caulis (Tiepishihu) by Fourier transform infrared spectroscopy and two-dimensional correlation spectroscopy. J. Mol. Struct..

[26] Sohng W., Park Y., Jang D., Cha K., Jung Y.M., Chung H. (2020). Incorporation of two-dimensional correlation analysis into discriminant analysis as a potential tool for improving discrimination accuracy: Near-infrared spectroscopic discrimination of adulterated olive oils. Talanta.

[27] LI L., Ma G., Cheng Y., Zhu C., Xu M., Xu X. (2018). Study on Performance of Diatomite and Basalt Fiber Compound Modified Asphalt Based on DSR Test. Mater. Rep..

[28] Yu J., Luo X., Wu S., Zeng X., Cong P. (2007). Preparation and Properties of Flame-retarded SBS Modified Asphalt. China J. High Way Transp..

[29] Wang F., Zhang L., Yan B., Kong D., Li Y., Wu S. (2019). Diffusion Mechanism of Rejuvenator and Its Effects on the Physical and Rheological Performance of Aged Asphalt Binder. Materials.

[30] Xie S., Xu W., Su Y. (2020). The Analysis on Anti-aging Performance of Carbon Nanotubes/SBS Compound Modified Asphalt Based on DSR Test. Synth. Mater. Aging Appl..

[31] Zhao Y., Gu F., Huang X. (2011). Analysis on SBS Modified Asphalt Aging Fourier Transform Infrared Characterization Based on Spectroscopy. J. Build. Mater..

[32] Yao H., Dai Q., You Z. (2015). Fourier Transform Infrared Spectroscopy characterization of aging-related properties of original and nano-modified asphalt binders. Constr. Build. Mater..

[33] Horch M., Schoknecht J., Wrathall S.L.D., Greetham G.M., Lenz O., Hunt N.T. (2019). Understanding the structure and dynamics of hydrogenases by ultrafast and two-dimensional infrared spectroscopy. Chem. Sci..

[34] Qu L., Chen J.-B., Zhou Q., Zhang G.-J., Sun S.-Q., Guo Y.-Z. (2016). Identification of authentic and adulterated Aquilariae Lignum Resinatum by Fourier transform infrared (FT-IR) spectroscopy and two-dimensional correlation analysis. J. Mol. Struct..

[35] Chen H., Li C., Sun J., He Q. (2019). Effect of interactive aging considering ultraviolet on rheological properties of SBR modified asphalt and analysis of the aging mechanism. J. Chongqing Univ..

[36] Wu W., Jin Y., Wu J., Zhang Y. (2019). Analysis of lignin modified asphalt by infrared spectrum. J. Jiangsu Univ. (Nat. Sci. Ed.).

[37] Shen T., Fu C., Mu Z., Zhang Z., Bao M. (2007). Two-Dimensional Infrared Correlation Spectroscopy Analysis. J. Univ. Jinan Sci. Technol..

